# Syntactic Processing in the Aging Brain: Neural Reorganization, Cognitive Scaffolding, and Implications for Language Resilience

**DOI:** 10.3390/brainsci16030251

**Published:** 2026-02-24

**Authors:** Xinmiao Liu, Shengqi Wu

**Affiliations:** 1 National Research Centre for Foreign Language Education, Beijing Foreign Studies University, Beijing 100089, China; 2 School of English for Specific Purposes, Beijing Foreign Studies University, Beijing 100089, China

**Keywords:** syntactic processing, aging, neural reorganization, cognitive scaffolding, language resilience

## Abstract

**Highlights:**

**What are the main findings?**

- The core left-lateralized frontotemporal language network remains resilient in older adults.
- Age-related change is reflected in heterogeneous network connectivity and increased engagement of non-core regions.

**What are the implications of the main findings?**

- Language resilience is maintained through a graded and condition-dependent adaptation of neural resources.
- Distinguishing domain-specific compensation from domain-general scaffolding is critical for interpreting neural reorganization.

**Abstract:**

Objectives: Although behavioral studies suggest that syntactic comprehension is relatively preserved in healthy aging, the underlying neural mechanisms remain a subject of intense debate. This review aims to synthesize neuroimaging and electrophysiological evidence to clarify how the aging brain reorganizes to maintain language resilience. Methods: A systematic search was conducted across multiple databases such as PubMed and Web of Science. Twenty-three relevant empirical studies meeting our inclusion criteria were identified. The synthesis focused on regional activation patterns, functional connectivity, and temporal dynamics during syntactic processing in older adults compared to younger controls. Results: The review revealed four key findings. First, the core left-lateralized frontotemporal language network remains resilient during syntactic processing in older adults. Second, age-related changes in functional connectivity within the core network are heterogeneous, with evidence for both reduction and preservation. Third, right-hemisphere homologues are increasingly recruited, but its functional significance is condition-dependent, serving both compensatory and non-compensatory roles. Fourth, older adults increasingly engage domain-general cognitive control regions, such as the dorsolateral prefrontal cortex and pre-supplementary motor area, to support syntactic processing under high cognitive loads. Conclusions: On the basis of these findings, we propose the Graded Compensation and Cognitive Scaffolding (GCCS) model which posits that language resilience is maintained through a graded and condition-dependent adaptation of neural resources. This study critically evaluates the current literature and highlights the need for more methodologically rigorous studies to better understand the effects of aging on syntactic processing and its neural basis. Given the limited number of eligible studies, the findings of this review should be interpreted with caution. More well-powered, longitudinal research is needed to uncover the trajectory of neural reorganization during syntactic processing in older adults.

## 1. Introduction

Syntactic processing is an essential component of human language comprehension [1]. Although both human and other animal species are capable of mapping sound to meaning [2], human language stands out for its remarkable productivity. Humans are able to generate infinite sentences using a finite set of lexical items [3]. Given its central role in language comprehension, syntactic processing has long attracted the attention of neuroscientists. The neural implementation of syntax provides critical insights into language networks in the brain and ongoing debates on functional specialization. The aging brain offers a unique opportunity to examine these neural correlates, as age-related changes in domain-general cognition enable us to test whether syntactic processing depends on a language-specific neural network or flexibly reorganizes under cognitive constraints [4,5]

Research on syntactic processing in the aging population dates back to the 1980s. Early studies provided behavioral evidence for the age-related changes in syntactic abilities using online or offline paradigms [6,7,8]. Behavioral findings generally indicate that the comprehension of simple sentences is preserved in older adults, but they tend to have difficulty in comprehending complex sentences with long-distance dependencies or temporary ambiguity [9,10]. These difficulties are often attributed not to impairment of core syntactic mechanisms, but to declines in domain-general cognition, such as working memory [10]. However, behavioral evidence alone cannot locate the underlying sources. It remains an open question about whether these difficulties reflect syntactic decline or the lack of effective neural compensation.

In recent years, a growing body of neuroimaging and electrophysiological research has investigated the neural basis of syntactic processing in older adults [11,12,13,14]. However, the findings remain mixed. Some studies suggest the syntactic performance in aging is supported by compensatory recruitment of domain-general cognitive systems, whereas others argue that it reflects the inherent resilience of the linguistic module, which is the relative stability of language-specific neural mechanisms supporting syntax [11,12,13,14]. For instance, an fMRI study by Grossman et al. reported increased recruitment of working memory regions in older adults under high syntactic demands [15]. Tyler et al. [12] observed a shift toward bilateral frontotemporal activation in older adults during low-demand tasks, a pattern interpreted as evidence of functional reorganization of the language network in response to gray matter loss. Yet, some studies challenge this view, suggesting that the syntax system remains largely intact. Campbell et al. [16], for instance, showed that the frontotemporal syntax system is resilient to aging. The activations in other regions merely reflect the reliance on domain-general resources under task demands. PET studies, such as those by Caplan et al., further stressed that age effects may stem from reduced processing speed rather than syntax loss [11]. Inconsistencies across neuroimaging and electrophysiological studies appear to be systematically related to methodological factors. Task characteristics and individual differences have also been shown to modulate age-related neural effects [17]. As a result, similar neural activation patterns have often been interpreted in different ways across studies. The neural correlates of syntactic processing in the aging brain remain unclear. To address this issue, the present study synthesizes evidence from neuroimaging and electrophysiological studies to clarify age differences in the neural organization during syntactic processing. By integrating findings across methodologies, we aim to identify underlying neural patterns that enable the brain to maintain language resilience despite biological aging. In the present review, the terms language resilience and preservation are used in an operational sense, referring to both behavioral and neural levels. Behaviorally, they denote relatively preserved performance in sentence comprehension tasks in older adults. Neurally, they refer to the maintenance of language network engagement despite age-related changes.

## 2. Materials and Methods

### 2.1. Literature Search

A systematic literature search was conducted to identify neuroimaging and electrophysiological studies examining syntactic processing in older adults. Searches were performed in Web of Science, PubMed and Google Scholar. All records were accessed up to 2 December 2025. Boolean combinations included terms related to syntax (e.g., syntax, syntactic processing, sentence processing, grammatical processing), aging (e.g., aging/ageing, older adults, elderly), and neurophysiological methods (e.g., fMRI, PET, EEG, ERP). Filters were applied to restrict results to human studies and to participants aged 65 years and above. We also performed two rounds of manual searches to identify studies not retrieved through database searches. A total of 395 studies were identified in the initial search.

### 2.2. Inclusion and Exclusion Criteria

Studies were included if they met the following criteria: (1) the study focused on an aging population; (2) they employed experimental paradigms that explicitly manipulated syntactic structure; (3) they used neuroimaging or electrophysiological methods to measure neural responses during syntactic processing; and (4) they reported neural outcomes relevant to regional activation, lateralization, network engagement, or temporal dynamics of syntactic processing. Studies were excluded if (1) they did not examine syntactic processing; (2) they did not focus on aging; (3) they involved non-human participants; (4) the full text is not available; (5) the articles were written in languages other than English; or (6) they were review articles, books, book chapters, editorials or conference proceedings. In this study, syntactic manipulation is defined as experimental contrasts in which the primary differences between conditions are structural properties such as word order, syntactic ambiguity and syntactic violation. When syntactic and non-syntactic factors were both involved, studies were included only when there was a clearly defined syntactic contrast and non-syntactic factors were matched across conditions. The purpose of this study is to review neural signatures of syntactic processing as elicited by syntactic contrasts, and how syntactic processing is modulated or supported by domain-general cognition under varying task demands in aging. Effects from syntactic contrast are treated as evidence relevant to syntactic processing, whereas effects from task-demand contrasts are interpreted as domain-general support.

After removal of duplicates, titles and abstracts were screened independently by two reviewers to assess eligibility. Full texts of potentially relevant studies were then screened against the inclusion criteria. Inter-rater agreement was relatively high for title and abstract screening (Cohen’s κ = 0.76). Disagreements were resolved through a consensus meeting between the two reviewers. As this study focuses on the syntactic process at the sentential level, studies were thus not included if their primary manipulation was semantic or pragmatic factors. For example, the two studies by Wlotko and Federmeier [18], and by Wlotko et al. [19], were excluded because they focused on semantic predictive processing at the sentential level, rather than syntactic processing per se. Oh et al.’s study was also excluded as it explored animacy effects on sentence processing [20].

**Figure 1:**
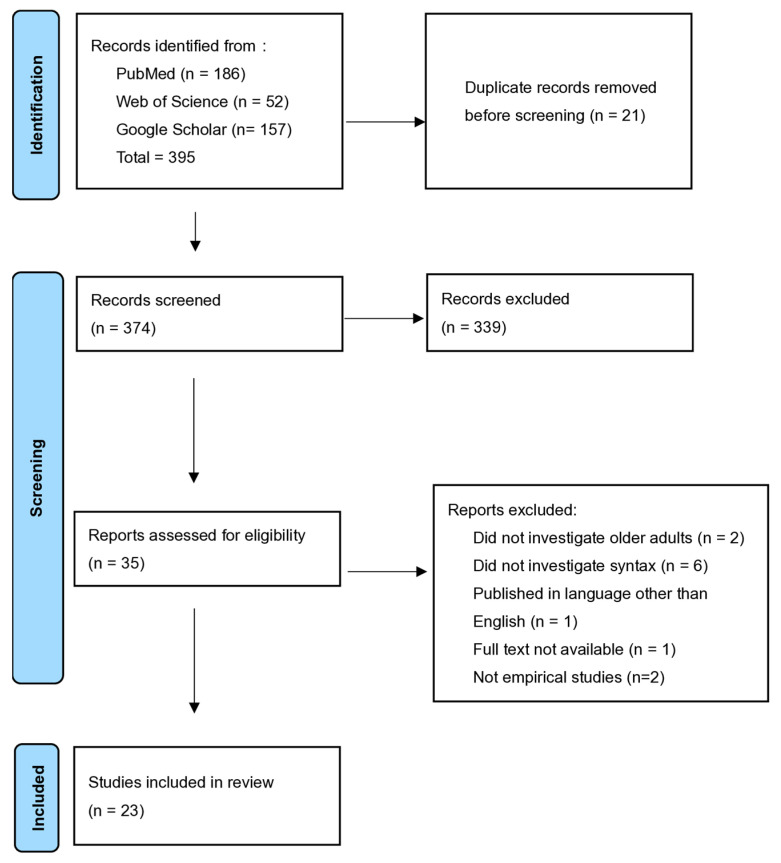
PRISMA diagram showing the process of the literature search.

A total of 23 studies were included in the final corpus. A PRISMA flow diagram summarizing the selection process is provided in Figure 1. This review followed key PRISMA principles to enhance transparency in the literature search, study selection, and quality assessment, although a review protocol was not registered. The PRISMA checklist used in this study is provided in Table S1. Specific search strategies can be found in Table S2.

### 2.3. Quality Assessment

This study adopted Wadsley and Ihssen’s modified version of the Effective Public Health Practice Project (EPHPP) Quality Assessment Tool for Quantitative Studies to evaluate the risk of bias [21,22]. The assessment criteria included selection bias, study design, participant characteristics and related methodological factors. Each item was assigned one point when the criterion was clearly and adequately satisfied (“Yes”), and zero points when it was not or insufficiently described (“No” or “Unclear”). For example, for the selection bias item “Are the individuals selected to participate likely to be representative of the target population?”, a score of one was assigned if the study clearly defined the target population, and reported recruitment procedures and eligibility criteria indicating the sample is representative. A score of zero was assigned when the recruitment was likely to yield a highly selected sample, or when the recruitment information was not clearly described. The item scores were summed to yield a global quality score ranging from 0 to 10. Based on these scores, the studies were classified into low quality (below 4 points), moderate quality (5–7 points), or high quality (above 8 points). Quality assessment was conducted independently by two reviewers. The inter-rater agreement is relatively high (κ = 0.84). Any difference was resolved through consensus discussion.

Data extraction was guided by a coding scheme (Table 1). Extracted data included study characteristics, syntactic manipulations, neurophysiological methods, patterns of brain activation, connectivity measures, and behavioral outcomes relevant to syntactic processing. Data extraction was performed independently by two reviewers. Inter-rater agreement was 87.4%. Any discrepancies were resolved through consensus discussion. Key characteristics and findings were tabulated to facilitate analysis. To assess potential reporting bias, we compared the reported outcomes in the final publications with their original study protocols where available. When critical methodology details or statistics were missing, we prioritized the information provided in the main text or consulted Supplementary Materials.

### 2.4. Analytical Framework for Synthesis

As the included studies employed diverse tasks, syntactic manipulations, and neural measures, we did not conduct a meta-analysis. Instead, we synthesized the findings using a narrative approach. An analytical framework was developed prior to data synthesis, following principles of structured qualitative and narrative synthesis [23]. This approach involves developing an initial conceptual framework, grouping studies into synthesis categories based on recurring patterns, and comparing findings across studies to identify convergence and sources of divergence. Findings were organized along four dimensions, each reflecting a different aspect of age differences in syntactic processing. First, we examined age-related changes within canonical left-hemisphere (LH) language regions, such as the inferior frontal and temporal cortices. The purpose was to explore whether these regions remain stable or show evidence of decline with aging. Second, findings related to hemispheric engagement were summarized, with particular attention to changes in lateralization and increased bilateral activation during syntactic processing. Third, we evaluated evidence for the recruitment of domain-general cognitive control networks. We used this category to evaluate whether older adults rely more on non-linguistic support systems, especially under high task demands. Fourth, electrophysiological evidence was synthesized to identify changes in the temporal dynamics of syntactic processing. Specifically, we examined differences in the amplitude, latency, or scalp distribution of ERP components associated with syntactic analysis such as the P600. This framework allowed us to compare results across studies to identify consistent patterns as well as sources of divergence in the neural organization. The studies were mapped to the four dimensions based on the reported neural outcomes and task characteristics. The analysis was first conducted by the first reviewer and then checked by the second. Any discrepancy was resolved through discussion. The robustness of the synthesis was assessed qualitatively by examining the consistency of findings across different modalities and samples. Potential sources of heterogeneity were explored through subgroup comparisons based on study characteristics.

## 3. Results

### 3.1. Overview of Included Studies

The included studies employed diverse methodologies. Nine studies used functional magnetic resonance imaging (fMRI) to examine spatial patterns of brain activation. Nine studies used event-related potentials (ERP), providing high temporal resolution for tracking the time course of syntactic processing. One study adopted magnetoencephalography (MEG). Four studies combined two techniques such as fMRI and ERP. All studies adopted cross-sectional designs. Most studies were conducted in the United States and the United Kingdom. Other studies are from Germany, Mexico, and Canada. Quality assessment indicated that all included studies were rated as being of moderate or high quality. See Table A1 in Appendix A for the studies included in this review and Table S3 for the results of quality assessment.

### 3.2. Age-Related Changes in Core Language Regions

A central question addressed in this study is whether aging is associated with functional changes within canonical language regions supporting syntactic processing. Among the eleven fMRI studies, nine reported that the left-lateralized frontotemporal language network (FTN) remained activated in older adults during syntactic processing [12,15,16,17,24,25,26,27,28]. This network includes the left inferior frontal gyrus (LIFG, BA 44/45) and left temporal regions. Evidence for the preservation of these language regions is relatively consistent across the included studies. Given the heterogeneity of paradigms across studies, preservation of the core language system was evaluated primarily based on the presence of comparable contrast effects. Preservation is defined as the maintenance of syntactic contrast effects within language regions in older adults. In a large-scale fMRI study, Campbell and colleagues found that the FTN was highly responsive to syntactic manipulation in both older and younger adults. No age differences were observed in responsivity to syntactic processing demands or functional connectivity within the FTN [16]. Mack et al. [24] examined the processing of English passive sentences using a sentence–picture verification task and found that in both age groups, passives elicited greater activation in left temporo-occipital regions and the bilateral inferior frontal gyrus (IFG) compared with active sentences. Apart from the left frontotemporal regions, the study suggested that the right IFG may be involved in the effortful syntactic reanalysis associated with task demands. No significant age-related differences were found in activation patterns in any brain region [24].

Converging evidence comes from two studies using more naturalistic paradigms. Davis et al. [17] compared natural listening conditions with explicit tasks and showed that during passive comprehension, older adults engaged the same left frontotemporal regions as younger adults. Age-related differences emerged only when additional task demands were imposed [17]. Vogelzang et al. [25] examined how older adults processed syntactically complex sentences in German. Twenty-two older adults listened to canonical and non-canonical sentences while completing a picture-matching task under both single-task and dual-task conditions. Under low load, object-initial sentences increased engagement in left frontal and temporal regions, whereas adjunct-initial sentences increased activation in occipital and right superior frontal regions. Higher load increased responses to simple sentences, but responses to complex sentences remained largely unchanged. Notably, this study did not include younger controls. Peelle et al. reported the only clear evidence of an age-related decline in the activation of the left ventral inferior frontal gyrus. During comprehension of relative clauses under challenging listening conditions, older adults showed weaker activation in the left IFG/anterior insula, and stronger activation in regions such as the left middle frontal gyrus (MFG) and right superior frontal gyrus (SFG) [29].

Across studies, evidence for age-related changes in functional connectivity within the FTN is mixed. While three studies reported reduced connectivity in the left-lateralized FTN in older adults [12,26,29], one study reported no significant age differences [16]. Antonenko et al. [26] examined the processing of relative clauses using a sentence–picture matching task. They found that functional connectivity among left-hemisphere syntactic regions was reduced in older adults. In younger adults, strong connectivity within a left-lateralized network predicted better performance, whereas in older adults, this connectivity was no longer associated with behavioral success. Instead, older adults showed increased connectivity with additional right-hemisphere and frontal control regions [26]. Greater inter-hemispheric coupling involving the right IFG and angular gyrus was associated with poorer comprehension performance in older adults [26]. Peelle et al. [29] found that older adults showed a significant reduction in functional coordination between regions, which predicts the decline in processing efficiency. Campbell et al. [16] provided the only evidence showing that functional connectivity of the frontotemporal network was stable across age groups. Task performance in older adults was more strongly predicted by interactions between the language network and domain-general control systems than by activity within the language network [16].

Evidence from diffusion tensor imaging (DTI) suggests that age-related changes are organizational rather than regional in nature. Antonenko et al. [26] demonstrated that, in younger adults, syntactic performance was closely associated with the integrity of dorsal white matter pathways, particularly the superior longitudinal fasciculus. These pathways are known to support rapid hierarchical syntactic processing. In older adults, however, this relationship was greatly reduced. Their performance depended more on ventral pathways such as the uncinate fasciculus [26].

### 3.3. Hemispheric Lateralization Changes

Another recurring theme in the literature concerns age-related changes in hemispheric engagement in syntactic processing. While younger adults typically show strongly left-lateralized activation in classical language regions, findings in older adults are mixed. Within the fMRI studies, six studies reported reduced left lateralization in older adults, and three reported that the FTN remains lateralized despite their advancing age, particularly when task demands are low [16,17,24]. Campbell et al. found that during the natural listening task, the left-lateralized frontotemporal language network remained dominant in older adults, with no significant increase in right-hemisphere activation [16]. Similarly, Mack et al. observed bilateral IFG activation in both younger and older adults during passive sentence processing, but found no age-related shift toward greater right-hemisphere reliance [24]. Davis et al. also showed that during natural listening, both age groups engaged primarily left-hemisphere regions, with no evidence of increased bilateralization in older adults [17].

In contrast, six studies provide evidence for reduced left lateralization in aging. Tyler and colleagues examined hemisphere-by-age interactions, and found that while activation in the left IFG was comparable across age groups, older adults showed significantly greater activation in the right IFG, resulting in a more bilateral activation pattern [12]. Lee et al. [27] similarly reported broader bilateral engagement in older adults. However, the right-hemisphere activity was located mainly in regions such as the insula and pre-SMA and was most evident during general sentence processing rather than for syntactic complexity contrasts. Peelle et al. also observed increased involvement of frontal regions outside the core language network in older adults, particularly under challenging listening conditions [29].

The functional interpretation of bilateralization varied across studies. Five studies suggest that recruiting additional resources in the RH helps older adults maintain language comprehension despite structural decline [12,15,28,29,30]. In Tyler et al.’s study [12], two lines of evidence supported the compensatory role of right hemisphere (RH) activities. First, greater recruitment of the right IFG was associated with preserved performance in older adults, with no age-related differences observed in word position effects. Second, RH compensation was supported by the relationship between gray matter density and neural activity. The increased functional activity in the RH frontotemporal regions was associated with atrophy in corresponding left-hemisphere regions. The authors argued that the recruitment of RH regions more likely reflected syntactic processing rather than reliance on domain-general resources, as working memory measures were not correlated with task performance. Lee et al. [27] reported that activity in several RH regions was negatively associated with performance. However, this relationship was found only in correct trials, which shows that RH activation did not contribute to poor performance. Instead, activity in the right prefrontal cortex might facilitate language performance in older adults.

However, two studies suggest that increased bilateral activity may reflect a loss of efficiency or specialized processing, rather than a successful effort to maintain performance [26,31]. Both studies reported negative associations between bilateral activation patterns and behavioral performance in older adults. Antonenko et al. [26] found that increased connectivity from left BA 44 to the right IFG and right angular gyrus was negatively associated with performance in older adults. This indicates that inter-hemispheric functional coupling may be detrimental under certain conditions. Antonenko et al. [31] found that stronger inter-hemispheric functional coupling between prefrontal areas was associated with worse behavioral outcomes in older adults, suggesting it reflects a loss of functional specialization or a lack of inhibition. These findings indicate that bilateralization is not uniformly beneficial and, in some cases, may reflect reduced processing efficiency rather than successful compensation.

### 3.4. Recruitment of Domain-General Control Networks

Beyond changes within the language network, eight studies report age differences in the recruitment of domain-general cognitive control regions. These effects are most evident in tasks that place high demands on working memory, attention, or executive control. The domain-general networks reported in the included studies span a broad set of regions including dorsal lateral/rostral PFC, superior frontal regions, dorsal frontal and parietal regions, insula, supplementary motor area (SMA)/pre-SMA and posterior cingulate.

The most frequently reported age-related increases in activation occur in the dorsolateral prefrontal cortex (dlPFC) and SFG. These regions are key components of the fronto-parietal control network, which supports decision-making, response selection, and working memory. Seven studies reported the activation of these areas in older adults. Davis et al. reported significant age-related changes in the activation of bilateral PFC during a task that did not involve language comprehension [17]. Further analyses showed that gray matter density (GMD) in several prefrontal regions mediated the increased activation of the bilateral frontal networks in older adults [17], suggesting that these activation changes may be attributable to gray matter atrophy. Campbell et al. [16] compared the neural activity during natural listening comprehension and explicit task-based conditions and found that several domain-general networks were recruited during explicit tasks. These included the multiple demand network (MDN; bilateral middle/inferior frontal gyri, superior medial frontal region, and marginally the left intraparietal sulcus), opercular network (OPRC; anterior cingulate cortex and bilateral anterior insula), basal ganglia, bilateral motor, and a negatively loading default mode network (DMN). Within-network connectivity during the explicit task declined with age and with gray matter concentration loss in some domain-general networks, particularly the MDN. Between-network connectivity also declined with age and gray matter concentration for several pairs during the task condition. Critically, only connectivity between the FTN and these domain-general networks predicted overt task performance. Stronger FTN-MDN connectivity was associated with better performance [16].

Another domain-general system recruited more strongly in older adults involves medial frontal regions, including the SMA and pre-SMA. These areas are found to be activated in older adults in four studies. Peelle and colleagues manipulated the speech rate to increase auditory processing demands and found that older adults experienced more difficulty under fast speech conditions. Older adults showed more activation in medial and dorsal frontal regions, including the superior frontal gyrus and SMA [29]. These regions are commonly associated with response inhibition, monitoring, and executive control. Peelle et al. also reported increased involvement of the inferior parietal cortex, interpreted as reflecting heightened working memory demands required to maintain and integrate fast auditory input [29]. Consistent with this account, Lee et al. [27] distinguished between syntactic complexity and general sentence processing and found that age-related increases in right-hemisphere activation, particularly in pre-SMA, were most evident during general sentence processing. This pattern suggests that older adults may rely on these regions to maintain processing efficiency during challenging tasks, highlighting their role in response monitoring and sequencing.

In addition to medial frontal regions, the insula and cerebellum are also recruited in older adults during syntactic processing tasks. These areas are commonly associated with salience detection and task-related emotional regulation. These regions were found to be activated in six studies. Lee et al. [27] observed increased activation in the right insula and pre-SMA, which could reflect monitoring and task control during sentence processing. Bilateral insular activations contributed to the overall reduction in hemispheric lateralization in older adults, as quantified by laterality index analyses across atlas-based ROIs. Activation in these areas was related to task performance. Lee et al. also reported cerebellar activation, suggesting a potential role for the cerebellum in supporting timing, motor control, and the integration of cognitive resources during more complex sentence processing tasks [27].

Recruitment of domain-general networks is particularly evident under conditions of high task demand, such as dual-task paradigms. Vogelzang et al. [25] investigated neural mechanisms of complex sentence processing in older adults. Using auditory presentation, they manipulated word order to create canonical and non-canonical structures, presented in single-task (comprehension only) and dual-task (comprehension plus WM) conditions. Domain-general cognitive control elements were more strongly engaged under dual-task load, during which syntactic complexity effects were reduced or eliminated. Task contrasts revealed increased activation in the right MTG and left MFG (BA 6), a dorsal frontal executive region often part of multiple-demand networks for working memory and task demands. Additional activation in the left supplementary motor cortex and brainstem activations further suggested the involvement of general sequencing, planning, and effort-related processes when syntactic demands competed with cognitive loads.

Given that hearing loss and increased auditory effort in older adults can strongly drive frontal and multiple-demand network recruitment and potentially mimic patterns of cognitive scaffolding, we examined whether hearing acuity was measured or controlled across the included auditory studies. We found that hearing acuity was measured or controlled in the majority of auditory studies included in this review. Only one auditory study did not report objective hearing assessment [26]. When restricting interpretation to studies that controlled for hearing, the observed patterns remain largely consistent. This suggests that the recruitment of domain-general networks is unlike to be driven solely by auditory efforts.

### 3.5. Temporal Dynamics of Syntactic Processing

Electrophysiological evidence provides complementary insight into the temporal dynamics of syntactic processing in aging. Across studies, older adults often show relatively comparable behavioral performance under low-demand conditions, but exhibit systematic changes in ERP components when processing demands increase or syntactic operations become more complex. A review of ERP studies reveals limited evidence for early components such as left anterior negativity (LAN) in older adults. To date, only Alatorre-Cruz et al. reported a clear LAN in older adults under conditions involving local morphosyntactic agreement violations [32]. In this study, participants read Spanish sentences containing gender agreement violations between determiners and nouns. Under these conditions, both younger and older adults showed a LAN in the 300–500 ms time window over left anterior scalp sites. However, the LAN observed in older adults was reduced in amplitude and was more sensitive to working memory load, particularly as syntactic distance increased. In two other studies, LAN was observed in younger adults but was greatly reduced or absent in older adults [13,19]. These findings suggest that early automatic structure-building processes may be weakened with aging. The LAN has traditionally been linked to early, fast, and local syntactic operation, such as feature checking. However, the interpretation and reliability of the LAN remain debated [33]. It shows considerable variability across studies, can overlap with the N400 component, and is highly task-dependent [33]. Many studies employed sentence materials involving longer dependency, competition, or integrative processing demands [13,19], which may reduce the likelihood of detecting LAN in older adults.

The P600, a late positive component typically associated with syntactic processing, was reported in all included studies. Its scalp distribution differed between age groups. Among the included ERP studies, six studies explicitly examined topography and reported age-related changes in scalp distribution [13,14,34,35,36,37]. Four studies described the P600 using its conventional posterior or centro-parietal distribution. Reduced lateralization was reported in five studies, with P600 effects becoming more bilaterally distributed in older adults compared with the left-dominant patterns in younger adults. Specifically, Leckey and Federmeier [13] observed a clear anterior shift in the P600 in older adults, with activity extending from posterior sites to more frontal regions. Similar frontal or more broadly distributed P600 patterns were observed in other studies [34]. This indicates that monitoring and control processes may play a larger role in syntactic processing in older adults. Chen et al. combined visual half-field presentation of syntactic phrase violations with DTI, and found that both younger and older adults showed left-hemisphere P600 responses to grammatical violations, but only older adults elicited a reliable right-hemisphere P600 effect [37]. Critically, the magnitude of this right-hemisphere P600 effect was positively correlated with higher fractional anisotropy values in the splenium of the corpus callosum. No such association was found for the genu [37].

With respect to amplitude, findings were mixed. Two studies reported larger or more sustained P600 amplitudes in older adults under high processing demands at certain electrodes [38], whereas five studies found comparable amplitudes across age groups when behavioral accuracy was matched [39,40]. This variability appears to be task-dependent: larger amplitudes in older adults are more likely when tasks are demanding or require explicit judgments, whereas amplitude differences diminish when performance accuracy is comparable between groups. Zhu et al. [39] investigated syntactic processing of simple Chinese sentences and reported that P600 amplitude is comparable between age groups, but in older adults, larger P600 amplitude is associated with worse performance. Findings regarding P600 latency were also heterogeneous. Zhu et al. [39] found a significantly delayed P600 peak latency in older adults. Alatorre-Cruz et al. [32] found longer latency of the P600 component under high WM load conditions among older adults with a risk of cognitive decline. However, two studies reported preserved timing. Kemmer et al. found comparable onset and peak latencies across age groups [36], and Alatorre-Cruz et al. reported no significant group differences in P600b latency despite clear amplitude changes [38].

While most research focused on time-locked ERP components, Poulisse et al. [14] took a novel approach by examining age differences in EEG oscillatory activity using a minimal phrase paradigm. Participants listened to two-word sentences that were either correct or incorrect and judged their grammaticality. The study revealed increased theta, alpha, and beta power at the second word in older adults. A significant group difference was found in the alpha range. Given that alpha power has been linked to attentional control, resource allocation and inhibitory control [41,42], these findings suggest that older adults may rely more on cognitive control compared with younger adults.

## 4. Discussion

Previous research has documented age-related neural reorganization during syntactic processing, yet findings remain inconsistent regarding the nature of these changes. The present study aimed to clarify this issue by integrating evidence from neuroimaging and electrophysiological studies. In this section, we summarize key findings and their implications, discuss limitations of the current literature, and propose directions for future research.

### 4.1. Summary and Interpretation of Key Findings

A review of previous studies found that the left frontotemporal network is consistently engaged during syntactic processing in older adults, and most studies reported no significant age difference in mean activation within these regions. This finding indicates that the fundamental neural architecture supporting syntactic processing is not compromised by aging and remains responsive in older adults. However, this does not mean the network is functionally intact. For instance, compensation in some older adults could offset group differences, leading to no change in mean activation. In addition, older adults might adopt different processing strategies, or the findings could reflect task-related confounds, normalization procedures, or structural atrophy effects that are common in group-based fMRI analyses. Evidence from functional connectivity analyses reveal heterogeneous age-related changes in the coordination of this network. While several studies report reduced functional connectivity within the left frontotemporal network, one study observed largely preserved connectivity under low task demands. While the individual network nodes remain responsive, their ability to communicate and operate efficiently as an integrated system may be reduced in high load conditions. This finding aligns with studies showing that processing speed, neural timing, and long-range communication are particularly vulnerable to aging, even when basic representations remain intact [43].

This study further indicates that bilateralization in older adults is highly task dependent. Bilateral engagement occurs more frequently in explicit tasks with high cognitive loads, whereas naturalistic comprehension often continues to rely on the FTN. Whether increased RH activation in older adults reflects compensation or neural decline depends on anatomical location and task context. In the present study, RH recruitment is considered as compensatory when the following conditions are met: (i) RH activation shows a positive relationship with behavioral performance, and/or (ii) it is associated with reduced structural or functional integrity of homologous LH regions, and (iii) it emerges under high task demands. According to this definition, true compensation appears when recruitment of the RH homologues, such as the right IFG, is directly linked to preserved performance in the presence of LH structural or functional decline [12]. When increased activation is negatively associated with behavioral performance, RH recruitment is likely to reflect neural inefficiency or dedifferentiation. As shown by Grossman and colleagues [15,28], greater RH activation can be associated with poorer comprehension, suggesting it reflects a failure to efficiently engage the language network. We propose that the function of RH recruitment in sentence comprehension may be governed by a threshold of efficiency. In high-performing older adults or during tasks of moderate complexity, RH resources may be flexibly recruited to maintain performance. Conversely, in the presence of severe FTN degradation or under extreme loads, RH recruitment may reflect a desperate but inefficient attempt to utilize domain-general cognitive resources. Bilateralization may therefore be viewed as a conditional response rather than a uniform marker of neural resilience. These observations highlight the need for caution when interpreting age-related changes in activation patterns through compensation frameworks. HAROLD (Hemispheric Asymmetry Reduction in Older Adults) [44] describes reduced hemispheric asymmetry as a common pattern in aging. Although this pattern has often been interpreted as compensatory in some of the literature, it is sometimes associated with neural dedifferentiation or reduced functional specialization in language processing [26]. CRUNCH (Compensation-Related Utilization of Neural Circuits Hypothesis) [45] posits that additional neural recruitment occurs to compensate for increased task demands. In the context of language processing, however, extra frontal activity can also occur when older adults rely on less efficient processing strategies [46,47]. This study cautions against interpreting increased activation as compensatory without clear evidence. The functional meaning of bilateralization depends on which regions are recruited and under what conditions.

Another major finding concerns the recruitment of domain-general cognitive control networks in older adults including multiple regions such as the dlPFC, SFG, SMA, pre-SMA, insula and cerebellum. The language network and domain-general control systems are functionally distinct [4]. Accordingly, recruitment of regions such as the insula or pre-SMA is better interpreted as the engagement of general cognition. Activation of domain-general control networks represents a strategic reorganization toward cognitive scaffolding, which is different from the compensation via RH homologues. The evidence synthesized here suggests that aging triggers a transition from language-specific processing to cross-network synergy. Domain-general regions do not appear to engage in syntactic parsing. Instead, they provide auxiliary support to manage increased cognitive burden. For example, the dlPFC supports the maintenance of complex structures in working memory, and pre-SMA facilitates response monitoring and sequence management [27]. This pattern is consistent with the scaffolding hypothesis [48], which posits that top-down executive control supports comprehension as specialized modules lose efficiency. As domain-general resources are shared across cognitive domains, their recruitment can sometimes introduce bottlenecks. As Vogelzang et al. suggested, when domain-general resources are exhausted by dual-task demands, the compensatory mechanism collapses [25].

The findings from ERP studies provide a temporal perspective on neural reorganization in aging. The early LAN component was found to be reduced or absent in older adults across several studies. Given that the functions of the LAN remain debated, its attenuation in aging may reflect age-related changes in early neural responses to syntactic processing. In addition, systematic age-related differences were observed in the P600, a component associated with syntactic integration, reanalysis, and repair. Its distribution often shifts toward more frontal and bilateral patterns in older adults, mirroring the fMRI findings of increased fronto-parietal and RH engagement. P600 latency findings were mixed. While some studies reported delayed P600 peak latencies in older adults, others found preserved timing, indicating that temporal dynamics are task-dependent. The present study indicates that early neural processes are vulnerable to age, and later, controlled processes become more distributed in topography. The temporal dimension of processing remains underrepresented in current models of aging. By integrating these dimensions, future theories can provide a more precise account of how syntactic processing is maintained or disrupted across the lifespan.

### 4.2. The Graded Compensation and Cognitive Scaffolding (GCCS) Model

In this section, we propose a Graded Compensation and Cognitive Scaffolding (GCCS) model of syntactic processing in aging to account for neural reorganization in the aging brain. According to this model, age-related neural reorganization should be considered as a graded and condition-dependent adaptive process, rather than a unitary shift in lateralization or resource allocation. The model involves three levels of adaptation. At the primary level (Level 1), aging leads to condition-dependent changes in processing efficiency of the FTN. Rather than large-scale deactivation, this phase is characterized by heterogeneous changes in network coordination. This may manifest as weakened functional connectivity and temporal desynchronization under high-load conditions, and preservation under less demanding conditions. While the language network remains operational, it becomes less fluent and more vulnerable to high load. At the second level (Level 2), the brain attempts to remediate inefficiency through the conditional recruitment of RH homologues, particularly the right IFG. The GCCS Model posits that RH involvement is compensatory only under specific conditions. When LH structural integrity is relatively preserved and task complexity is moderate, the RH may provide effective support. When these thresholds are exceeded, RH activation ceases to be supportive, and instead signals neural dedifferentiation or processing inefficiency. The most robust adaptation occurs at the third level (Level 3), which involves the strategic engagement of domain-general control systems, particularly the multiple-demand network. These regions do not perform syntactic parsing directly. Instead, they provide top-down cognitive support. The dlPFC supports the active maintenance of incomplete syntactic dependencies in working memory [49]. The anterior cingulate monitors conflict during ambiguity resolution [50]. The pre-SMA aids in the sequential ordering and timing of linguistic representations [51].

In the model, compensation and scaffolding represent two different neurobiological strategies that differ in function. Compensation (Level 2) refers to functional remediation within the linguistic domain. Scaffolding (Level 3) refers to strategic support from domain-general systems. While compensation is to preserve the nature of the computation, scaffolding represents a fundamental shift in strategy, transforming linguistic operations into resource-intensive, effortful cognitive tasks. Adaptations at Level 2 and 3 represent two partially independent response pathways. RH recruitment primarily reflects rebalancing within the language system under syntactic challenge, whereas recruitment of domain-general control reflects responses to memory, decision, and perceptual demands. Which pathway is engaged depends on task characteristics.

This model provides powerful explanations for the empirical contradictions identified in this review. Different studies, using different tasks and samples, may tap into different levels of this adaptive hierarchy. A natural listening task may reveal only changes at Level 1. For a moderately complex sentence judgment task, compensatory right-IFG activity at Level 2 might be detected in high-performing seniors. A demanding dual-task paradigm may induce strong reliance on Level 3 scaffolding, which can eventually collapse under extreme load, as seen in Vogelzang et al. [25]. Based on this model, the following predictions can be made. First, GCCS predicts an interaction between task demand, LH structural integrity and behavioral performance. Under moderate demand and relatively preserved LH integrity, IFG recruitment in the right hemisphere should correlate positively with behavioral performance such as accuracy. Under higher demand or reduced integrity, the recruitment of the domain-general network should become the stronger predictor of performance. Under extreme load, GCCS further predicts a ceiling effect, where the domain-general network no longer has a supportive role. Second, GCCS predicts different neural signatures between naturalistic comprehension and explicit judgment paradigms. Under naturalistic listening or reading, age effects should mainly reveal Level 1 changes, with limited engagement of domain-general control. Under moderately demanding syntactic comprehension tasks such as sentence judgment, older adults are predicted to show increased recruitment of the right IFG. Under the dual-task paradigms that impose high cognitive demands, the model predicts increased recruitment of the domain-general network, with behavioral effects more closely tied to control measures. In addition, GCCS also predicts different connectivity and ERP signatures for each adaptive level under increasing load. At Level 1, increased task demand is expected to weaken functional connectivity within the FTN and to attenuate early ERP responses of syntactic analysis. At Level 2, compensatory RH recruitment should be marked by increased functional connectivity between the right IFG and the left FTN. This should be accompanied by a shift in later positivity toward a more bilateral or frontal distribution. At Level 3, with increased domain-general scaffolding, we expect increased connectivity between the domain-general network and the FTN. In addition, later ERP responses are expected to show a more consistently bilateral or anterior distribution under high load.

While earlier models such as HAROLD, CRUNCH and STAC-r provided the important foundation for aging neuroscience, the GCCS model refines these frameworks by introducing functional specificity and hierarchical boundaries tailored for language. It clarifies that previous studies were divided possibly because researchers often look at different levels of the adaptive hierarchy. By distinguishing these levels, GCCS inspires future researchers to examine an individual’s neural strategy based on their neural reserve and task characteristics.

In a recent large-scale fMRI study, Jain et al. [52] showed that language networks remain engaged across the adult lifespan, while exhibiting selective reorganization in connectivity patterns. This pattern is consistent with the GCCS view that neural reorganization in aging is graded and contingent on processing demands. The study found that age-related changes are most evident in frontal and frontotemporal pathways, whereas temporal connectivity is largely stable, particularly under resting-state conditions [52]. This anatomical asymmetry suggests that network coordination may emerge unevenly across regions within the FTN. Although this finding is based on evidence about global language ability, rather than syntactic process alone, it suggests that changes at Level 1 may not be uniform across the entire left frontotemporal system. Instead, changes in network coordination might be spatially heterogeneous. Further work is needed to explore whether similar asymmetrical patterns characterize syntactic processing.

### 4.3. Critical Evaluation of the Literature

This study highlights several methodological issues that should be addressed in future investigations. The first concern is the heterogeneity of linguistic structures used to operationalize syntactic processing. The included studies examined a wide range of constructions from simple active–passive contrasts to more complex structures such as relative clauses and garden path sentences. These structures differ substantially in the cognitive operations they recruit, making it difficult to attribute neural patterns only to syntax. For example, relative clauses, the most frequently used structures in previous studies, place heavy demands on working memory and inhibitory control [53,54]. Therefore, this type of structure often leads to activation in regions such as the dlPFC and inferior parietal regions. Ambiguous sentences often require reanalysis, conflict monitoring, and selection among competing interpretations [55,56,57]. Processing such sentences is likely to trigger the cingulo-opercular network. Neural patterns observed in such studies may reflect stimulus-driven cognitive load. Without differentiating hierarchical syntactic operations from the executive demands imposed by particular sentence types, researchers risk misidentifying domain-general task support as language-specific compensation. Future research should employ more refined linguistic paradigms to isolate syntactic operation from general cognitive load.

A related issue is that previous studies define syntactic processing at different analytical levels. Some investigations quantify overall engagement of the language system, for example by contrasting sentence comprehension, with a baseline condition. Others focus on syntactic complexity, comparing complex and simple sentences, or ambiguous and unambiguous constructions. These two approaches might tap into partially different neural systems. The former likely triggers the engagement of the core frontotemporal language network. In older adults, these regions typically show stable or even increased activation, which is often interpreted as evidence for the resilience of syntax. However, such activation may also reflect semantic integration and lexical procedural retrieval. The latter approach targets incremental structure building and revision, which are likely to engage the LIFG. When age differences are found in paradigms that mix syntactic, semantic, and executive demands, it becomes impossible to determine whether the differences reflect syntactic decline, increased reliance on control processes, or simply vulnerability to task difficulty. Some claims about age-related changes in syntactic processing may in fact be based on experimental designs that do not isolate syntax with sufficient rigor.

In addition, many experimental tasks require explicit judgment or decision-making. Such tasks can engage domain-general control processes, which are known to be particularly vulnerable to aging. For example, sentence–picture matching tasks required participants to maintain the entire sentence in working memory, compare it with a visual display, and make a judgment [24,26]. Evidence from several studies demonstrates that age effects are highly sensitive to such task demands. Davis et al. [17] showed that bilateral frontal overactivation in older adults emerged only during overt tasks, and disappeared under natural listening conditions. Similar issues were reported by Campbell et al. [16]. These findings suggest that some reported neural differences may reflect task-related processing strategies rather than syntactic mechanisms.

Even when task design is appropriate, inferences about age-related neural mechanisms are sometimes based on indirect evidence. Many fMRI studies rely on univariate activation contrasts. While such analyses identify regions involved in processing, they do not directly indicate how networks operate. Age-related changes in syntactic processing are often interpreted in terms of increased or decreased activation in specific regions, yet such differences do not necessarily reflect changes in underlying mechanisms. As shown in several studies reviewed here, older adults can display comparable levels of activation in core language regions while still showing reduced functional connectivity and coordination. However, relatively few studies [16,26] have employed connectivity-based analyses that can more directly assess network-level changes. Without such approaches, conclusions about neural reorganization remain tentative. Future research should shift from mapping regions of activation toward quantifying the topological efficiency of the syntactic network.

### 4.4. Suggestions for Future Research

To provide clearer insights into how aging affects syntactic processing, future research needs to address several critical issues in methodology. Below we outline some recommendations for future investigations.

First, future studies should adopt experimental designs that can clearly separate syntactic processing from other cognitive functions. One promising approach involves using model-based and naturalistic paradigms. Computational predictors such as syntactic surprisal or structure-building complexity can be used to avoid forcing end-of-sentence judgments. For instance, syntactic surprisal models, which track complexity dynamically during sentence comprehension [58], could be adopted to assess how older adults process different levels of syntactic difficulty. Recent work has shown that during naturalistic language, WM-related predictors can be tested under strict surprisal controls inside the language-selective cortex [59]. Such approaches can reduce the confounds associated with decision or monitoring, making it easier to identify which aspects of syntactic processing are sensitive to aging, and under what computational demands.

Given the methodological heterogeneity, it is difficult to compare findings across studies. Future studies would benefit from greater alignment between analytical levels. Studies focusing on mean activation, functional connectivity, and structural connectivity often draw conclusions at different explanatory levels. These measures should be integrated within the same experimental framework, allowing activation, connectivity, and structure–function coupling to be jointly evaluated. This is particularly important for aging research. To enable cross-modal synthesis, we suggest that future studies adopt convergent representational frameworks across neuroimaging modalities. For example, comparable anatomical or functional definition of language networks would facilitate integration of fMRI, DTI and electrophysiological measures.

Methodological rigor can also be strengthened by improving how language regions are defined in fMRI studies. Most aging studies rely on group-level anatomy or coarse atlas-based ROIs to identify language areas. This might be problematic because aging brains differ widely in cortical atrophy, sulcal anatomy, and normalization accuracy. What is labeled as the left IFG or left temporal cortex in group analyses may represent different neural tissue across age groups, making it difficult to determine whether observed differences reflect functional changes or simply ROI mismatch. A reliable alternative is subject-specific functional localization. In this approach, regions are defined individually for each participant using an independent localizer contrast, such as sentences versus nonwords. These functionally defined regions can then be used to assess syntactic effects. This method has been validated in neurolinguistics and shown to increase sensitivity, functional specificity, and cross-participant comparability [60,61]. With subject-specific localization, we could identify the different ways by which individuals maintain language resilience. In addition, within-subject designs should be considered to combine structural MRI, functional MRI, and electrophysiological measures. By assessing these modalities in the same participant sample, researchers could directly link structural integrity, network coordination, and temporal dynamics to provide a comprehensive view of the structural and functional networks involved in the syntactic analysis during aging. Given the high level of individual variability in the aging brain, such designs are critical for identifying the trajectory of age-related changes in syntactic processing.

Future work should place greater emphasis on network-level analytic approaches. Although connectivity-based analyses have provided some of the most informative findings to date [16,26], existing evidence remains limited in scope and methodology. Most studies have adopted coarse measures of functional connectivity or independent component analysis. Only a few studies have directly examined how interactions between the language network and domain-general control networks change with age. Future studies should make greater use of connectivity models, such as psychophysiological interaction (PPI), dynamic causal modeling (DCM), or Granger causality analyses, to test directional influences between regions. In addition, multivariate pattern analysis and graph-theoretic network metrics can reveal the changes in representational structure and network efficiency that are not detectable through univariate contrasts. Such analyses would allow us to specify precisely which connections break down, which new connections emerge, and under what conditions these changes support or hinder comprehension.

ERP research likewise requires methodological refinement. Rather than treating the presence or absence of components such as LAN as indicators of syntactic function, future studies should model age-related variability in timing and dynamics. Approaches that are less dependent on strict time-locking, such as time-frequency analyses and neural tracking methods, offer promising alternatives. EEG has been shown to track hierarchical linguistic units without relying on canonical component windows [62], and intracranial recordings have revealed detailed dynamics of phrase structure building [63]. These analyses would provide a richer account of how the temporal dynamics of syntax change with aging.

Finally, more attention should also be paid to individual differences and linguistic coverage. Rather than treating older adults as a homogeneous group, future studies should report narrower age bands or treat age continuously. Individual differences in hearing ability or processing speed should be assessed to explore how these factors mediate neural patterns. Variability in life experience, neural integrity, and strategy will change scaffolding across individuals [45]. Studies that consider these moderators can provide a fine-tuned perspective into how language processing changes with aging. In addition, most studies on syntactic processing in aging have focused on English, which may limit the generalizability of their findings to other languages. Languages with rich morphology, free word order, or pro-drop structures may exhibit different patterns of aging-related neural changes in syntactic processing. Future studies should incorporate typologically diverse languages to determine whether current findings reflect universal principles or language-specific properties.

This study is not without limitations. First, the study focuses only on externally driven syntactic processing which is syntactic computation triggered by auditory or visual language input. However, inner speech was not considered. Previous studies show that inner speech engages overlapping areas with those involved in syntactic processing [64]. As inner speech does not rely on external sensory input, it may reduce perceptual demands and, in some contexts, lessen the need for domain-general control. Therefore, it provides an effective approach to disentangle syntactic computation from perceptual and domain-general control components. Such an approach enables us to better understand the neural mechanisms underlying syntactic processing in aging. Future studies could integrate inner speech paradigms in both cross-sectional and longitudinal designs, and evaluate whether exercising inner speech helps to preserve language performance in older adults. Another limitation is that one study was not included due to the lack of full text access, which may introduce availability bias. Future reviews could attempt to obtain full-text versions of inaccessible articles, or incorporate abstract-based sensitivity analyses. In addition, only studies published in English were included in this review, which may introduce language bias. Future studies should consider including relevant work published in other languages such as Korean. Such strategies would help reduce potential availability bias and provide a more comprehensive synthesis of the literature.

## 5. Conclusions

The present study shows that the aging brain undergoes complex neural reorganization during syntactic processing. Existing evidence suggests that age-related changes in syntactic processing are characterized by: (1) a relative preserved core frontotemporal language network; (2) heterogeneous changes in functional connectivity within the core network, with evidence for both reduction and preservation; (3) conditional recruitment of RH homologues, particularly the right IFG, serving both compensatory and non-compensatory roles; and (4) increased strategic reliance on domain-general cognitive control regions, including the dlPFC, anterior cingulate, and pre-SMA. To account for these adaptations, we propose the Graded Compensation and Cognitive Scaffolding (GCCS) model, which provides a unified framework to explain how the brain hierarchically shifts between specialized modules and general-purpose systems to maintain language resilience. Findings from the reviewed studies highlight the role of domain-general control regions in supporting sentence comprehension in older adults. Future research could explore whether cognitive training or non-invasive neuromodulation techniques such as tDCS and TMS targeting these regions may help mitigate age-related declines in language processing. However, given the limited number of eligible studies, the findings of this review should be considered tentative. More well-powered, longitudinal studies are needed to understand the trajectory of neural reorganization during syntactic processing in older adults.

## Figures and Tables

**Table 1:** Coding scheme.

Domain	Coding Description
Study Information	Authors and year of publication; empirical or review; design (cross-sectional, longitudinal)
Participants	Sample size; demographic details; exclusion criteria
Task Characteristics	Type of task; type of structures
Neuroimaging/Electrophysiology	fMRI, MRI, PET, ERP, MEG, DTI
Brain Activation Patterns	Activation in left IFG, MTG/STG, and related frontotemporal regions; recruitment of right hemisphere and other regions
Electrophysiological Signatures	Characteristics of ERP components; scalp distribution
Connectivity Measures	Intra- and inter-hemispheric connectivity patterns; integrity of white matter tracts
Behavioral Outcomes	Performance accuracy; response latency
Age-related Differences	Increased, decreased, preserved, or shifted activation; correlations between neural measures and performance

## Data Availability

Not applicable to this article as no new data were created or analyzed.

## Supplementary Materials

The following supporting information can be downloaded at: https://www.mdpi.com/article/10.3390/brainsci16030251/s1, Table S1: PRISMA 2020 Checklist [65]; Table S2: Database-specific Search Strategies; Table S3: Quality Assessment Results for the Included Studies.

## Appendix A

**Table A1:** Studies included in this review.

Authors	Journal	Year	Sample	Method	Regions	Key Findings
Davis et al. [17]	Neuropsychologia	2014	50 participants (age range: 20–86)	fMRI	FTN, opercular, fronto-parietal, and bilateral prefrontal cortex (PFC)	Frontotemporal activity during natural listening is stable. Bilateral PFC activity increases with age only during explicit tasks.
Lee et al. [26]	Aging Brain	2022	38 old (66 years)	fMRI	Bilateral frontotemporal network, right fronto-parietal cortex, cerebellum and SMA	Older adults recruit right-hemisphere resources (insula, pre-SMA, cerebellum) more than young adults during sentence processing.
Tyler et al. [12]	Cerebral Cortex	2009	14 young (23.9), 44 old (67.4)	fMRI	Left frontotemporal system, right frontotemporal regions, and bilateral intraparietal sulcus	Preserved syntactic performance is achieved through a shift from a left-lateralized system to a bilateral functional network. Aging weakened some frontotemporal connections.
Campbell et al. [16]	Journal of Neuroscience	2016	35 young, 37 middle-aged, 39 old	fMRI	FTN, auditory network, and multiple demand network	The functionality and internal connectivity of core FTN remains robustly stable. Between-network connectivity declines with age and gray matter loss.
Antonenko et al. [26]	NeuroImage	2013	21 old (70.4), 20 young (26.3)	fMRI, DTI	Left pars opercularis, SLF, UF, forceps minor, IFG	Young adults show stronger FC within left-lateralized networks. Young adults rely on dorsal SLF and elderly rely more on ventral UF.
Grossman et al. [15]	NeuroImage	2002	13 young (22.6), 11 old (63.5)	fMRI	Left posterolateral temporal, left IFG, left parietal, left premotor, and right temporal–parietal regions	Older adults show reduced recruitment of the left parietal cortex and more activation in the left premotor and dorsal left IFG. Seniors recruit the right parietal cortex for working memory challenges.
Vogelzang et al. [25]	Neurobiology of Language	2020	22 old (61 years)	fMRI	Left IFG, left temporal, occipital regions, right superior frontal, and left MFG/SMA	In older adults, distinct types of complexity (object- vs. adjunct-initial) elicit distinct neural patterns.
Mack et al. [24]	Brain Sciences	2013	14 young (24.9), 13 old (61.2)	fMRI	Bilateral IFG and the left temporo-occipital junction	Processing passives elicits activation in the bilateral IFG and left posterior temporal regions. No age difference was found in these activations.
Grossman et al. [28]	Brain and Language	2002	22 old: (good: *n* = 11, 63.5; poor: *n* = 11, 71)	fMRI	Left posterolateral temporal, left IFG, left prefrontal, right dorsal IFG, and left posterior cingulate	Seniors with poor comprehension show reduced recruitment of the core left perisylvian network and increased activation in left PFC, right dorsal IFG, and left posterior cingulate.
Peelle et al. [29]	Cerebral Cortex	2010	20 old (64.8), 20 young (22.4)	fMRI	Left IFG, middle frontal gyrus, left inferior parietal, left posterior MTG, and medial superior frontal gyrus	Older adults exhibit reduced left ventral IFG activity and functional connectivity, and the recruitment of non-core neural regions.
Leckey et al. [13]	Psychophysiology	2017	24 old (67 years)	ERP	Scalp-wide (26 electrodes) using a visual half-field paradigm to bias processing to the left or right hemisphere	Shift from strongly left-lateralized P600 in younger adults to a bilateral P600 pattern in older adults. Older adults’ P600 distribution is broader and encompasses anterior sites.
Zhu et al. [39]	Frontiers in Psychology	2018	26 young (21.9), 20 old (67.6)	ERP	Anterior and posterior regions (F, FC, P, CP electrodes) in both left and right hemispheres	Significant delay in P600 peak latency for older adults during syntactic processing. Comparable P600 amplitude between age groups.
Kemmer et al. [36]	Psychophysiology	2004	16 young (20), 16 old (69)	ERP	Scalp-wide (26 electrodes covering prefrontal, frontal, central, parietal, temporal, and occipital regions)	P600 amplitude and latency remain stable across ages for simple syntactic violations. P600 becomes less asymmetric and more frontal in older adults.
van Boxtel et al. [35]	Journal of Language and Aging Research	2023	18 old (69.6), 20 young (21.4)	ERP	Scalp-wide (64 electrodes); regions of interest including frontal, centro-parietal, and parietal clusters	N400/P600 attenuations are similar across age groups. Older adults show wider frontal distribution of P600 effects compared to the front-left distribution in younger adults.
Alatorre-Cruz et al. [32]	Clinical Neurophysiology	2019	44 old: risk group (*n* = 22, 67.7); control (*n* = 22, 65.2)	ERP	Scalp-wide (31 electrodes), with a focus on areas showing high theta absolute power in the left hemisphere	Older adults with excess theta activity show smaller amplitudes and longer latencies of P600a under high WM load.
Alatorre-Cruz et al. [38]	Frontiers in Human Neuroscience	2018	30 old (66.06), 30 young (25.7)	ERP	Anterior, central, and posterior electrode sites	Older adults show smaller amplitudes of LAN, P600a, and P600b specifically when working memory load increases.
Steinhauer et al. [34]	Neuroscience Letters	2010	13 old (65–80)	ERP	Midline electrodes (Fz, Cz, Pz) and lateral electrode arrays	Older adults elicit normal Closure Positive Shift and P600 effects. Frontal shift in the P600 distribution in the elderly.
Poulisse et al. [14]	Neuropsychologia	2020	19 young (21), 41 old (69)	ERP	Scalp-wide; clusters in frontal–central, left frontotemporal, and left parietal regions	Syntactic binding in seniors is associated with smaller increases in theta, alpha, and beta power. Young adults show larger increases for binding; older adults show smaller increases.
Lee & Lai [40]	Psychology and Aging	2024	34 young (23.9), 34 old (69.6)	ERP	Scalp-wide; specific focus on anterior vs. posterior mean amplitudes	P600 effect is preserved across ages. Older adults as a group lack the sustained anterior negativity.
Caplan et al. [11]	Human Brain Mapping	2003	13 old (73.4), 8 young (24.3), 9 old (75.2)	PET, ERP	Broca’s area, left inferior parietal, superior frontal gyrus, left superior parietal, and left inferior frontal lobe	Fast elderly adults activate left inferior frontal structures, similar to young adults. Slow responders shift recruitment toward parietal structures.
Antonenko et al. [31]	NeuroImage	2012	20 old (69.9 years)	fMRI, DTI	Bilateral IFG, the inferior parietal lobe	In older adults, performance correlates positively with white matter integrity in bilateral IFG. Inter-hemispheric functional coupling negatively predicts performance. Older adults exhibit altered synchronization in left IFG.
Chen et al. [37]	OSF Preprint	2022	35 old (69), 35 young (23)	DTI, ERP	Corpus callosum, orbitofrontal gyri, occipital lobes, posterior temporal and parietal regions, FTN	Older adults recruit RH P600 responses for syntactic violations. Magnitude of the P600 effect in seniors correlates with splenium microstructural integrity.
Kielar et al. [30]	Human Brain Mapping	2016	19 old (65.63), 21 young (24.55); 19 patients (65.1)	MEG	Left ventral frontotemporal, bilateral dorsal fronto-parietal and occipital regions	Older adults exhibit more extensive, bilateral responses to syntactic anomalies and decreased occipito-parietal activation.
