# Supplementary material for: Syntactic Processing in the Aging Brain: Neural Reorganization, Cognitive Scaffolding, and Implications for Language Resilience
Source: Brain Sci. 2026 Feb 24;16(3):251. doi: 10.3390/brainsci16030251 (PMC13024383; doi:10.3390/brainsci16030251)
Supplement: Supplementary file 1 [file brainsci-16-00251-s001.zip › Table S2.pdf]

Table S2. Database-specific Search Strategies

Database	Fields searched	Exact search query
**PubMed**	Title/Abstract; MeSH	("syntax"[Title/Abstract] OR "syntactic"[Title/Abstract] OR "syntax processing"[Title/Abstract] OR "syntactic processing"[Title/Abstract] OR "sentence processing"[Title/Abstract] OR "grammatical processing"[Title/Abstract] OR "grammaticality"[Title/Abstract])<br>AND<br>("aging"[MeSH Terms] OR "ageing"[Title/Abstract] OR "older adults"[Title/Abstract] OR "elderly"[Title/Abstract] OR "age-related"[Title/Abstract] OR "aging"[Title/Abstract] OR "aged"[MeSH Terms] OR "ageing"[Title/Abstract])<br>AND<br>("neuroimaging"[Title/Abstract] OR "functional magnetic resonance imaging"[Title/Abstract] OR "fMRI"[Title/Abstract] OR "PET"[Title/Abstract] OR "positron emission tomography"[Title/Abstract] OR "magnetic resonance imaging"[MeSH Terms] OR "EEG"[Title/Abstract] OR "electroencephalography"[Title/Abstract] OR "event-related potential*"[Title/Abstract] OR "ERP"[Title/Abstract] OR "electrophysiological"[Title/Abstract] OR "electrophysiology"[Title/Abstract])
**Web of Science (Core Collection)**	Title (TI); Abstract (AB)	(TI (syntax OR syntactic OR "syntactic processing" OR "sentence processing" OR "grammatical processing" OR grammaticality)<br>OR AB (syntax OR syntactic OR "syntactic processing" OR "sentence processing" OR "grammatical processing" OR grammaticality)

Database	Fields searched	Exact search query
		AND<br>(TI (aging OR ageing OR "older adults" OR elderly OR "age-related" OR aged)<br>OR AB (aging OR ageing OR "older adults" OR elderly OR "age-related" OR aged)<br>)<br>AND<br>TI (neuroimaging OR fMRI OR "functional MRI" OR "functional magnetic resonance imaging" OR PET OR "positron emission tomography" OR EEG OR electroencephalography OR ERP OR "event-related potential*" OR "event related potential*" OR electrophysiological)<br>OR AB (neuroimaging OR fMRI OR "functional MRI" OR "functional magnetic resonance imaging" OR PET OR "positron emission tomography" OR EEG OR electroencephalography OR ERP OR "event-related potential*" OR "event related potential*" OR electrophysiological)
**Google Scholar**	Full text (relevance-ranked)	("syntactic processing" OR "sentence processing" OR "grammatical processing") ("older adults" OR elderly OR aging OR ageing) (fMRI OR PET OR EEG OR ERP OR neuroimaging) -"child" -"children" -"pediatric" -"adolescent" -"developmental" -"childhood" -"young" -"youth" -"semantic" -"lexical" -"phonological"
